# Influence of Metamizole on Antitumour Activity of Risedronate Sodium in In Vitro Studies on Canine (D-17) and Human (U-2 OS) Osteosarcoma Cell Lines

**DOI:** 10.3390/biomedicines12081869

**Published:** 2024-08-15

**Authors:** Dominik Poradowski, Aleksander Chrószcz, Radosław Spychaj, Joanna Wolińska, Vedat Onar

**Affiliations:** 1 Department of Biostructure and Animal Physiology, Division of Animal Anatomy, Faculty of Veterinary Medicine, Wroclaw University of Environmental and Life Sciences, Kożuchowska 1, 51-631 Wrocław, Poland; 2 Department of Fermentation and Cereals Technology, Faculty of Biotechnology and Food Science, Wroclaw University of Environmental and Life Sciences, J. Chełmońskiego 37, 51-630 Wrocław, Poland; 3 Faculty of Veterinary Medicine, Wroclaw University of Environmental and Life Sciences, C.K. Norwida 25, 50-375 Wrocław, Poland; 4 Osteoarchaeology Practice and Research Centre & Department of Anatomy, Faculty of Veterinary Medicine, Istanbul University-Cerrahpaşa, 34320 Avcılar, Istanbul, Türkiye

**Keywords:** risedronate sodium, metamizole, osteosarcoma, in vitro study

## Abstract

The availability of metamizole varies greatly around the world. There are countries such as the USA, UK, or Australia where the use of metamizole is completely forbidden, and there are also countries where this drug is available only on prescription (e.g., Greece, Italy, Spain, etc.) and those in which it is sold OTC—over the counter (e.g., most Asian and South American countries). Metamizole, as a drug with a strong analgesic effect, is used as an alternative to other non-steroidal anti-inflammatory drugs, alone or in combination with opioid drugs. Risedronate sodium is a third-generation bisphosphonate commonly used in orthopaedic and metabolic diseases of the musculoskeletal system, including hypercalcemia, postmenopausal osteoporosis, Paget’s disease, etc. The aim of this study was to check whether there were any pharmacological interactions between metamizole and risedronate sodium in in vitro studies. Cell viability was assessed using the MTT method, the number of apoptotic cells was assessed using the labelling TUNEL method, and the cell cycle assessment was performed with a flow cytometer and propidium iodide. This was a pilot study, which is why only two cancer cell lines were tested: D-17 of canine osteosarcoma and U-2 OS of human osteosarcoma. Exposure of the canine osteosarcoma cell line to a combination of risedronate sodium (100 µg/mL) and metamizole (50, 5, and 0.5 µg/mL) resulted in the complete abolition of the cytoprotective activity of metamizole. In the human osteosarcoma cell line, the cytotoxic effect of risedronate sodium was entirely eliminated in the presence of 50 µg/mL of metamizole. The cytoprotective and anti-apoptotic effect of metamizole in combination with risedronate sodium in the tested human and canine osteosarcoma cell lines indicates an urgent need for further in vivo studies to confirm or disprove the potential dose-dependent undesirable effect of such a therapy.

## 1. Introduction

The coexistence of humans and dogs in the same environment, and the similarities in their biology, in the etiopathogenesis and histological structure of osteosarcomas, as well as in factors predisposing them to the disease (e.g., high body weight), allow for comparative in vitro studies to be carried out in established canine and human osteosarcoma cell lines. Due to the aforementioned similarities, the WHO classification of osteosarcoma in dogs and humans is very similar [1,2]. Based on the type of focus, type of matrix, location and degree of malignancy, and finally their histological structure, osteosarcomas can be classified as follows:

- primary or secondary;
- osteoblastic, chondroblastic, fibroblastic, or mixed;
- extraosseous or intraosseous (central, periosteal, paraosteal);
- low or high grade;
- classic, telangiectatic, or small cell.

Osteosarcoma (OSA) in dogs is diagnosed almost 27 times more often than in humans [3]. OSA prevalence in dogs accounts for over 80% of all diagnosed bone neoplasms [4,5,6]. However, in the population of dogs with a documented history of the disease, the percentage of animals with OSA is very low and usually does not exceed 10%, depending on the country [3,7]. In humans, the percentage of patients diagnosed with OSA is approximately 3% of the studied population [8]. Of all bone cancers in humans, it accounts for only 0.2% [9]. OSA in dogs and humans occurs most often in the long bones, less frequently in the flat or irregular bones, and according to an unwritten rule, “far from the elbow, near to the knee”. The extraosseous type of osteosarcoma can be found usually in the skin and subcutaneous tissues, liver, or lungs [10]. The male–female ratio in dogs is approximately 1:1 (it is more common in neutered than unneutered animals), and in humans it is 1.5:1 [6,9,11,12,13]. Presumably, a difference in body weight between canine and human males and females is an important factor influencing the disease aetiology and pathogenesis. In dogs, OSA is most often diagnosed in the age group of 7–10 years; sporadic cases of osteosarcoma occur in puppies and dogs over 10 years of age [6,14,15,16,17,18]. This disease most frequently affects individuals of giant and large breeds, such as the Great Dane, Rottweiler, Bernese Mountain Dog, Saint Bernard, German Shepherd, or Boxer [5,6,11,15]. In humans, the peak incidence occurs between the ages of 15–25, which may be explained by increased bone turnover accompanying the process of intense growth [9]. OSA is rarely diagnosed in children and, in older people around the age of 60, the cancer is secondary to Paget’s disease, overexposure to radiation, or bone infarction [9,19,20]. Etiopathogenesis of OSA in dogs and humans is not fully understood, but the most common predisposing factors include micro-injuries, overexpression of COX-2, or disorders in the synthesis of proteins such as ezrin or other proteins belonging to the ERM family. Clinical symptoms are also similar in both species, and these include lameness, fractures, swelling and pain of variable severity, and cachexia developing at advanced stages. It is worth emphasizing that osteosarcoma is not accompanied by any characteristic paraneoplastic syndromes, which may in some way improve the detection rate of this type of cancer. The treatment regimen for OSA in dogs and humans is fairly similar and involves [3]:

- amputation of the affected limb, resection of the affected tissues, or less traumatic limb-sparing methods;
- adjuvant chemotherapy using platinum derivatives (cisplatin and carboplatin), doxorubicin or, less commonly, etoposide;
- in humans, neoadjuvant chemotherapy is sometimes used, which involves administering chemotherapy not after but before surgery.

In addition to the cytostatic drugs, WHO recommends reducing the pain associated with the disease with strong analgesic and non-steroidal anti-inflammatory drugs, e.g., meloxicam, drugs from the coxib group (celecoxib), or metamizole (available in some countries) [21,22]. These drugs can be administered alone or in a combination with opioid analgesics (tramadol, butorphanol, or fentanyl). Metamizole, sometimes called dipyrone, is a non-steroidal anti-inflammatory drug belonging to the group of pyrazolones and the WHO “analgesic ladder” [23]. It was first discovered in 1920 by a German company, Hoechst AG, and was not mass-produced until 1922. Metamizole, which has an almost magical analgesic effect resulting from the inhibition of COX-2 and affecting kappa receptors [24], has numerous side effects occurring in dogs and humans, such as anaphylactic shock, agranulocytosis, broadly understood myelotoxicity, and aplastic anaemia. Due to its serious side effects, metamizole has been withdrawn from use in North American countries and some Scandinavian countries, while in Latin American countries, Asia, and some European Union countries, e.g., Poland, Hungary, or Romania, it is available without any restrictions [25,26].

Bisphosphonates are a group of active substances that have been used for a long time (the first substance from this group was synthesized over 40 years ago) for the treatment of metabolic and orthopaedic diseases in humans (including the adjuvant and palliative therapy of osteosarcoma), and are now more and more commonly used in dogs [27,28]. These substances reduce bone resorption, limit the ability of bone tumours to metastasize, inhibit neoangiogenesis, reduce the possibility of pathological fractures, and have an analgesic effect. In human medicine, bisphosphonates are used not only in the treatment of bone cancer, but also in multiple myeloma, breast, and prostate cancer metastases, as well as in the treatment of osteoporosis (especially postmenopausal), Paget’s disease, hypercalcemia, and cholecalciferol intoxication. Scientific research has shown that bisphosphonates have cytotoxic and pro-apoptotic properties towards cancer cells [27,29,30].

Previous studies using commonly used cytostatic drugs showed that metamizole at a concentration of 50 µg/mL has cytoprotective and anti-apoptotic effects. Therefore, metamizole may weaken the anticancer effect of the drugs used [31]. The authors want to present a similar study, this time regarding the combination of metamizole with risedronate sodium, which is often used in the treatment of osteosarcoma in both humans and dogs. The aim of this study was to prove this phenomenon in in vitro studies on canine and human osteosarcoma cell lines using the MTT test to assess cell viability, the terminal deoxynucleotidyl transferase dUTP nick end labelling (TUNEL) method to assess the percentage of apoptotic cells, and the cell cycle assay with propidium iodide.

## 2. Materials and Methods

### 2.1. Cell Cultures and Selected Drug Preparations

One canine and one human osteosarcoma cell line, D-17 and U-2 OS, respectively, were used in this study, both purchased from the American Type Culture Collection (Manassas, VA, USA). The cell lines were incubated in 25 cm^2^ and 75 cm^2^ culture flasks, in an incubator with a constant 5% flow of CO_2_ and a temperature of 37 °C (SANYO, model MCO-18AIC, Osaka, Japan). The D-17 line was maintained in Eagle’s Minimum Essential Medium (ATCC, Manassas, VA, USA), and the U-2 OS in McCoy’s 5A medium (ATCC, Manassas, VA, USA), supplemented with 10% foetal bovine serum (Sigma-Aldrich, Burlington, MA, USA), 4 nM of L-glutamine (Sigma-Aldrich, Burlington, MA, USA), 100 U/mL of penicillin, and 100 µg/mL of streptomycin (Sigma-Aldrich, Taufkirchen, Germany).

### 2.2. Selected Drugs

Metamizole (meta) (Sigma-Aldrich, Taufkirchen, Germany) and risedronate sodium (rd) (Sigma-Aldrich, Taufkirchen, Germany) were dissolved in the culture media. Table 1 presents the range of individual drug concentrations, selected based on their maximum serum concentration; in the case of both drugs, the type of solvent did not have a limiting effect on the selected concentrations.

From the tested combinations, we chose risedronate sodium at a concentration of approximately EC_50_ (Table 2) for both cell lines. For metamizole, the maximum concentration used was higher than that achieved in the canine plasma, to match that achieved in the human serum. This choice was intended to facilitate comparison of the obtained results (Table 3).

### 2.3. MTT Assay—Cell Viability Assessment

The D-17 and U-2 OS cells, at a concentration of 3 × 10^3^/100 µL suspended in the culture medium, were placed in 96-well culture plates (TPP, Trasadingen, Switzerland). After 24 h, the culture medium was replaced with a fresh one, and the tested compounds were added, alone and in combinations. The MTT (3-(4,5-dimethylthiazol-2-yl)-2,5 diphenyl tetrazolium bromide) test was performed in accordance with the standard PN-EN ISO 10993-5 [32]. Mitomycin C (a substance with known cytotoxicity) was used as a positive control, and clear culture medium was used as a negative control. The mean of the results obtained in four independent repetitions was presented as the final result.

### 2.4. TUNEL Method—Assessing the Percentage of Apoptotic Cells

**Figure 1:**
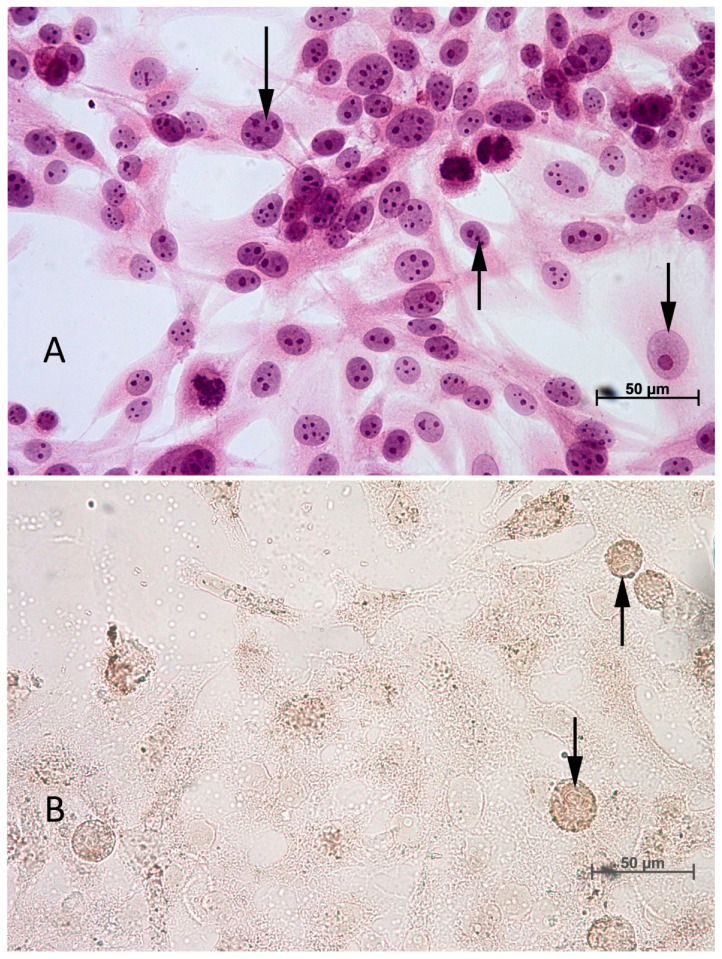
(**A**) Arrows point to normal cells. (**B**) arrows point to apoptotic cells.

The ApopTag^®^ Peroxidase In Situ Apoptosis Detection Kit (Merck Millipore, Darmstadt, Germany) was used in this study. The standard set consisted of the working strength TdT enzyme, an equilibration buffer, a stop/wash buffer, anti-digoxigenin peroxidase conjugate, and DAB peroxidase substrate. Cells of both tested lines, at a density of 2 × 10^4^ cells in 40 µL of dedicated culture medium, were placed on microscopic 10-well hydrophobic slides (Thermo Scientific, Waltham, MA, USA). After 24 h of incubation, the culture medium was removed and replaced with a new one, with dissolved drugs alone or in combinations (Table 1 and Table 3), for 72 h of incubation. Haematoxylin solution (1%) (Merck Millipore, Darmstadt, Germany) was used to counterstain the cell nuclei presented in Figure 1.

Finally, the slides were immersed for 30 s in 70% ethyl alcohol (Stanlab, Warsaw, Poland), then for 30 s in xylene (Stanlab, Warsaw, Poland), and coverslips were attached using DPX (Thermo Scientific, USA). Positively stained apoptotic cells (brown) were counted in five randomly selected fields of view (40×) under the Olympus BX53 optical microscope (Olympus, Tokyo, Japan), and the result was given as a percentage of all cells visible in the field of view. The final result was the average for five fields of view along with the standard deviation. The calculation and evaluation of the immunohistochemical reaction were performed by two experienced scientists.

### 2.5. Flow Cytometry—Cell Cycle Assessment

The cells from the D-17 and U-2 OS cell lines, at a density of 1 × 10^6^ cells in 2000 µL of the culture medium, were placed in sterile 6-well culture plates (TPP, Trasandingen, Switzerland). After 24 h, the culture medium was replaced with a fresh one, and the examined drugs, dissolved in the culture medium alone and in combinations, were added to each well (Table 1 and Table 3) for a period of 72 h. After the incubation, the cells were detached using a trypsin (0.25%)—EDTA (0.02%) solution (Sigma-Aldrich, Taufkirchen, Germany). Then, they were centrifuged and re-suspended in PBS. The cells at a density of 1 × 10^6^ were transferred to a centrifuging tube containing 1000 µL of PBS, and centrifuged for 5 min at 1200 rpm at 4 °C.

The cell pellet was gently mixed with 300 µL of PBS. The cells were fixed to the surface and their membranes were permeabilized with 700 µL of cold 70% ethyl alcohol, added dropwise. To prevent the formation of cell conglomerates, the samples were gently mixed while the alcohol was added. The cells were then incubated on ice for 1 h and, after centrifugation, the supernatant was removed and the cells were re-suspended in a mixture of 250 µL of PBS and 5 µL of RNAase A at a concentration of 10 mg/mL (Sigma-Aldrich, Burlington, MA, USA).

After a 60 min incubation at 37 °C, 10 µL of propidium iodide, at a concentration of 1 mg/mL (Sigma-Aldrich, Burlington, MA, USA), was added. The samples were analysed using a flow cytometer (FACSCalibur, Becton Dickinson, Franklin Lakes, NJ, USA) equipped with an argon laser with an excitation wavelength of 488 nm. The mean with the standard deviation of four independent repetitions was given as the final result.

### 2.6. Statistical Analysis

Statistical analysis was performed using the StatisticaPL 13.0 program (StatSoft, Kraków, Poland). In order to determine the normal distribution of the obtained results, the Shapiro–Wilk test was used. Significantly lower mean values of individual research objects than the control sample were demonstrated using the Dunnett test. In the figures, the horizontal lines within each box indicate the mean values. The lower and upper edges of the boxes represent the mean value with the standard deviation subtracted and added. The whiskers represent the minimum and maximum values. The significance level of the statistical tests was set at *p* = 0.05.

## 3. Results

### 3.1. EC_50_ Values

Poradowski et al. [31,33] published their own research presenting EC_50_ values for risedronate sodium and metamizole (Table 2).

### 3.2. Cell Viability

**Figure 2:**
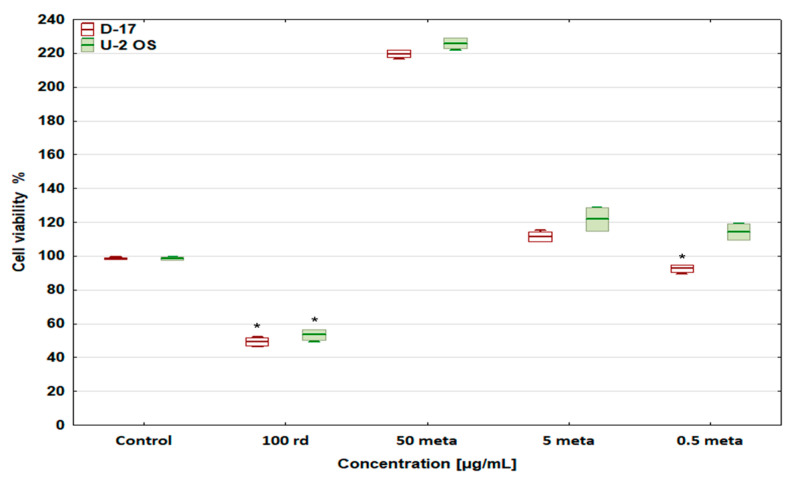
The effect of risedronate sodium and metamizole on cell viability of the D-17 canine and U-2 OS human osteosarcoma cell lines, * values below control in the Dunnett test.

The effect of risedronate sodium and metamizole alone, at the concentrations selected, for further studies on the viability of cells from the D-17 and U-2 OS osteosarcoma lines, is shown in Figure 2. Risedronate sodium, at the tested concentration, strongly reduced the viability of both cell lines [31], while metamizole exerted a potential concentration-dependent cytoprotective and proliferation stimulating effect [33].

**Figure 3:**
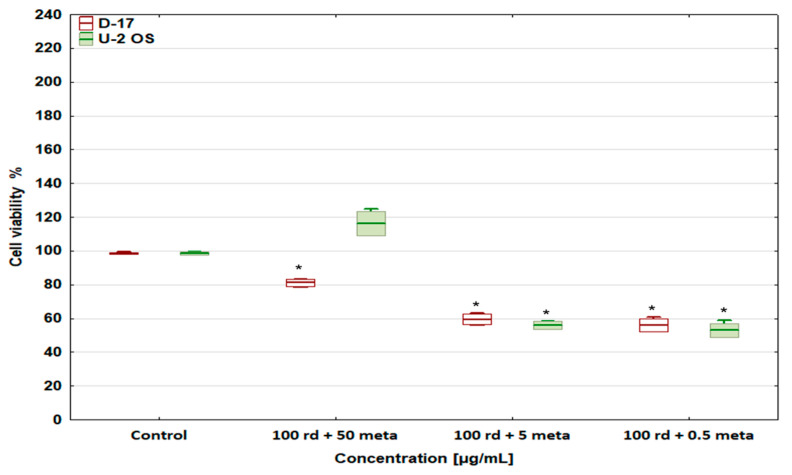
The effect of combinations of risedronate sodium and metamizole on cell viability in the canine D-17 and human U-2 OS osteosarcoma cell lines, * values below control in the Dunnett test.

Exposure of the canine osteosarcoma cell line to a combination of risedronate sodium at 100 µg/mL and metamizole at 50, 5, and 0.5 µg/mL resulted in a complete abolition of the cytoprotective activity of metamizole at these concentrations (Figure 3), and that was confirmed for metamizole alone (Figure 2). However, metamizole at 50 µg/mL weakened the cell viability-reducing effect of risedronate sodium at the tested concentrations compared with its use as a single drug (Figure 2). A reverse situation was observed in the human osteosarcoma line, as the cytotoxic effect of risedronate sodium was completely abolished by the presence of metamizole, and stimulated proliferation of the tested cells was also observed (Figure 3).

On the other hand, metamizole at lower concentrations (5 and 0.5 µg/mL) did not change the cytotoxic activity of the highest tested concentration of risedronate sodium (100 µg/mL) towards both neoplastic cell lines (Figure 3).

### 3.3. Percentage of Apoptotic Cells

**Figure 4:**
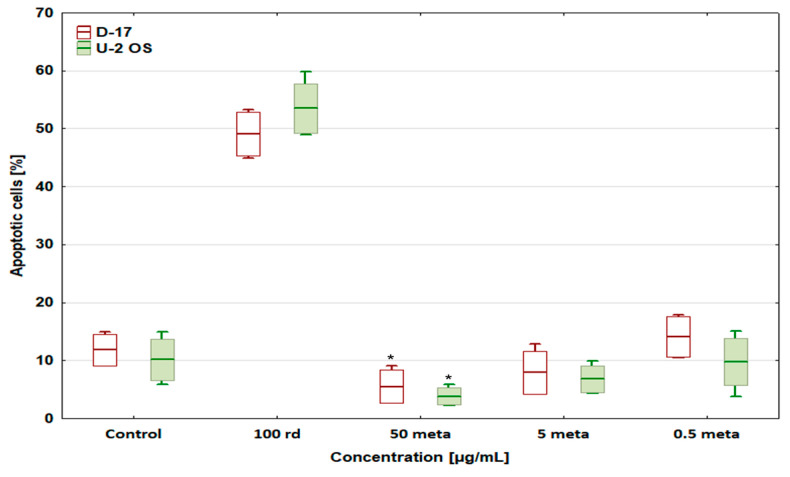
The effect of risedronate sodium and metamizole on apoptosis in the canine and human osteosarcoma cell lines, * values below control in the Dunnett test.

After the administration of risedronate sodium (100 µg/mL), the number of apoptotic cells in both tested lines (D-17 and U-2 OS) was significantly higher than in the control and reached 49.06 ± 3.77% and 53.5 ± 4.28% (Figure 4), respectively [33]. Metamizole shows a potential concentration-dependent cytoprotective effect. At 50 µg/mL, it lowered the percentage of apoptotic cells in both cell lines when compared to the control. At 5 and 0.5 µg/mL of metamizole, the number of apoptotic cells returned to a level close to that of the control, both in the D-17 and U-2 OS cell lines [31].

**Figure 5:**
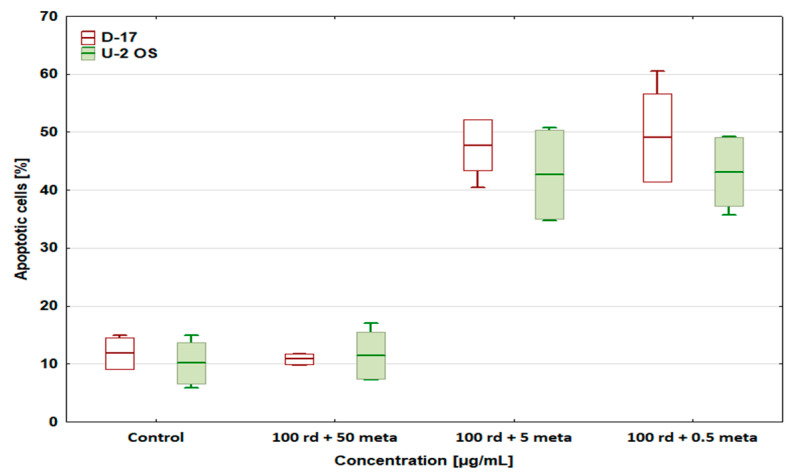
The effect of risedronate sodium and metamizole combinations on apoptosis in the canine D-17 and human U-2 OS osteosarcoma cell lines.

Metamizole at 50 µg/mL completely abolished the pro-apoptotic effect of risedronate sodium at 100 µg/mL, while lower concentrations of metamizole restored the percentage of apoptotic cells to a level similar to that obtained with risedronate sodium alone (47.72 ± 4.41 and 42.62 ± 7.67%, respectively, for 5 µg/mL, and 49.03 ± 7.65 and 43.15 ± 5.91%, respectively, for 0.5 µg/mL) (Figure 5). This was observed in both canine and human osteosarcoma lines.

### 3.4. Cell Cycle

**Figure 6:**
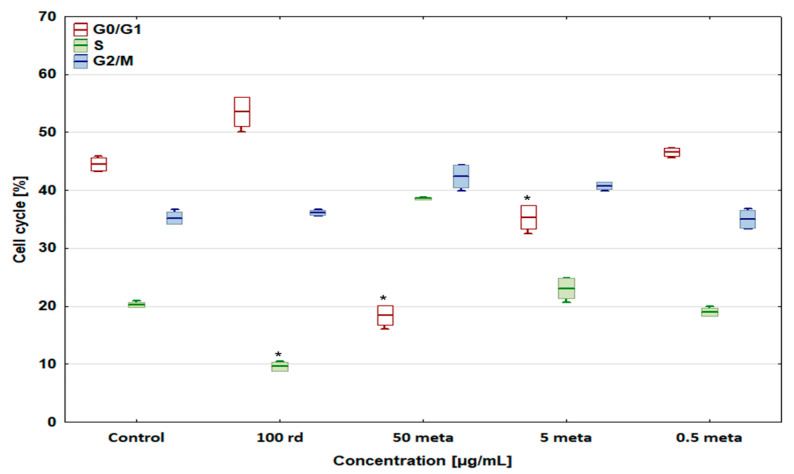
The effect of risedronate sodium and metamizole on the cell cycle in the canine osteosarcoma D-17 cell line, * values below control in the Dunnett test.

**Figure 7:**
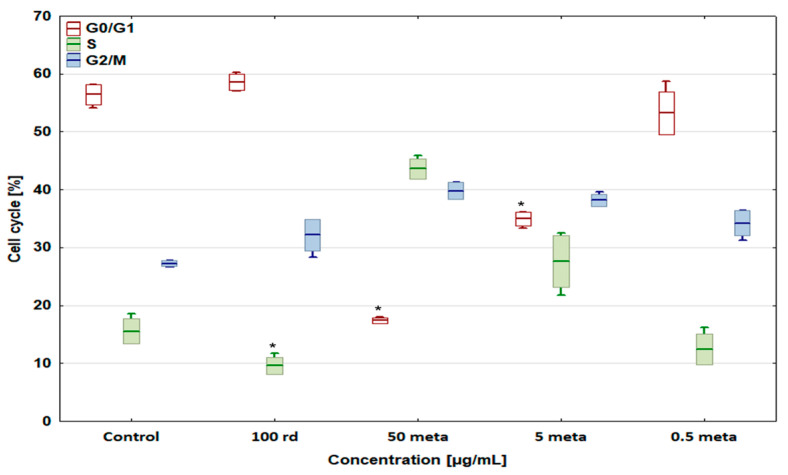
The effect of risedronate sodium and metamizole on the cell cycle in the U-2 OS human osteosarcoma cell line, * values below control in the Dunnett test.

Risedronate sodium slightly increased the percentage of cells in the G0/G1 phase (cell cycle arrest) to 53.60 ± 2.52% (D-17) and 58.58 ± 1.34% (U-2 OS), and significantly decreased the number of cells in the S phase in both cell lines (Figure 6 and Figure 7). Metamizole at 50 and 5 µg/mL decreased the number of cells in the G0/G1 phase and enhanced their number in the other phases. In the case of dog and human osteosarcoma cells treated with 0.5 µg/mL of metamizole, no significant differences were observed compared to the control group.

**Figure 8:**
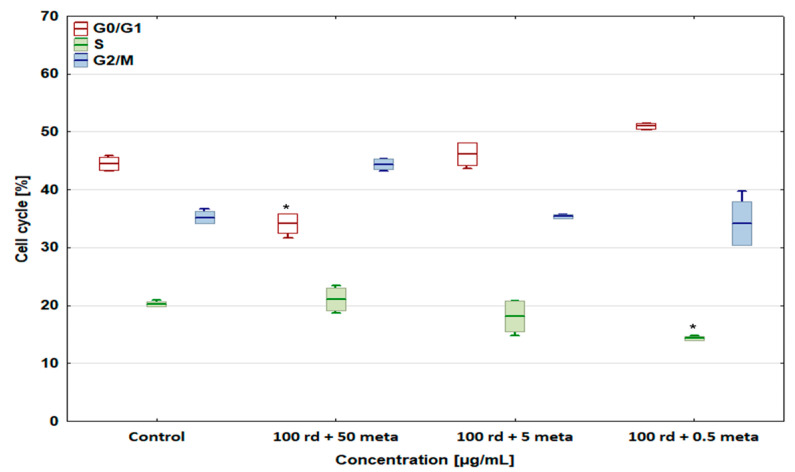
The effect of combinations of risedronate sodium with metamizole on the cell cycle in the canine osteosarcoma D-17 cell line, * values below control in the Dunnett test.

**Figure 9:**
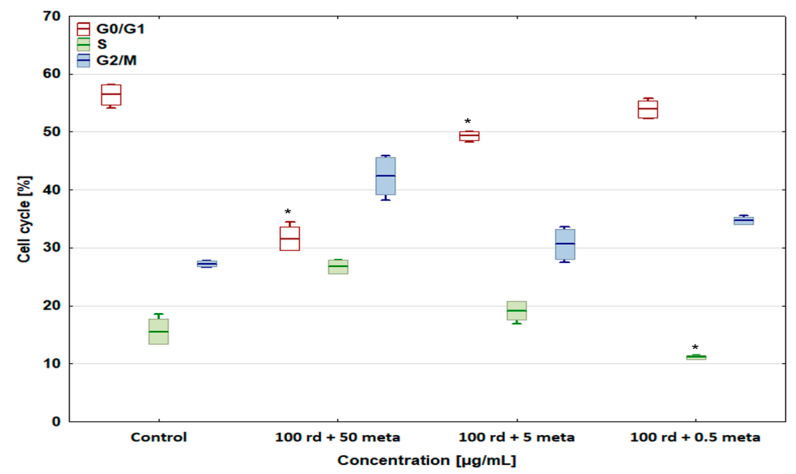
The effect of combinations of risedronate sodium with metamizole on the cell cycle in the U-2 OS human osteosarcoma cell line, * values below control in the Dunnett test.

The percentage of the D-17 and U-2 OS cells in the G2/M phase (cell cycle arrest) increased to 44.39 ± 0.9% and 42.39 ± 3.18% after exposure to the combination of 100 µg/mL of risedronate sodium + 50 µg/mL of metamizole compared with the control groups (Figure 8 and Figure 9), with a simultaneous significant decrease in the number of cells in the S phase. In the case of other metamizole concentrations, a concentration-dependent return to the values obtained in the control samples was observed.

## 4. Discussion

Our results indicate a need for further research and are of a preliminary and pilot nature. To confirm the hypothesis that metamizole has a cytoprotective effect and stimulates the proliferation of cancer cells, additional studies should be carried out involving normal cell lines and other commonly used cancer cell lines (e.g., Jurkat-T—cell leukaemia, MCF-7—breast cancer, LM-MEL-75—melanoma, etc.). Further investigations should focus on the cytoprotective effect of metamizole and its potential for stimulating neoplasm growth. Both human and animal cancer treatment should exclude any undesirable pharmacologic activity caused by routinely administered drugs to create fully effective protocols of use. Earlier studies showed that risedronate sodium (100 µg/mL) strongly reduced the cell viability of both tested cell lines, while metamizole (especially at a concentration of 50 µg/mL) proved its cytoprotection and stimulated cell proliferation [33]. Additionally, low concentrations of metamizole (0.5 and 5 µg/mL) did not provoke a similar effect, because the number of apoptotic cells decreased to the level of the control group [31]. These results proved the phenomenon of metamizole’s concentration-dependant pharmacological effect. Even though a high concentration of metamizole (above 50 µg/mL) has cytotoxic activity, the maximal drug concentration in serum does not exceed a value of 40 and 100 µg/mL in dogs and humans. Other studies have also proved that low concentrations of metamizole have not a toxic, but a cytoprotective effect [34].

This study was aimed at identifying the potential pharmacological interactions between risedronate sodium and metamizole, when administered together. Statistically significant differences were observed. The combination of 100 µg/mL of risedronate sodium with 50 µg/mL of metamizole caused the abolition of the pro-apoptotic activity of the first drug, while lower concentrations of the second drug in combination (100 µg/mL of risedronate sodium and 5 or 0.5 µg/mL of metamizole) did not show a cytoprotective effect and did not influence the cytotoxicity of risedronate sodium alone. Simultaneously, significant differences were observed between canine and human osteosarcoma cell lines when we look at the combination of 50 µg/mL of metamizole with 100 µg/mL of risedronate. In the canine osteosarcoma cell line, the addition of metamizole caused a slight weakening of risedronate sodium’s cytotoxic activity while, in the human osteosarcoma cell line, the risedronate sodium activity was completely abolished. These results seem to be not only interesting from a theoretical knowledge point of view, but could be especially important for the practical chemotherapy of bone cancer, especially in humans.

To confront in vitro results with the response of living organisms, and to describe and understand the mechanisms of metamizole’s activity, in silico tests and, finally, in vivo experiments should be carried out. Until now, few scientific reports on the cytoprotective effect of metamizole have been published [34,35]. Moreover, there are no reports on its possible interactions with bisphosphonates. The effects of risedronate sodium and metamizole combinations on the viability of canine osteosarcoma cells made us speculate on the potential cytoprotective effect of metamizole in vitro. This hypothesis was confirmed by Akgun et al. [31], in their studies on normal body cells. The complete abolition of the cytotoxic effect of sodium risedronate in the human osteosarcoma line is an important finding, as this could be dangerous for patients. This fact requires further attention as, in the case of a primary cell line derived from an intervertebral disc (*discus intervertebralis*), the cytoprotective effects of metamizole were also demonstrated. It can be explained by the fact that, despite numerous morphologic similarities, canine and human osteosarcomas are characterized by a different sensitivity to the combination of sodium risedronate and metamizole, especially at the highest tested concentrations. It is also worth emphasizing that the cytoprotective effect proven by Akgun et al. [31] occurs only at low concentrations of metamizole in the serum. The cytoprotective effect of metamizole at low concentrations is also supported by the research of Pompeia et al. [32], conducted on the HL-60, Jurkat, and Raji cell lines, in their studies using UV radiation, arachidonic acid, and cycloheximide. Metamizole shows its anti-apoptotic effect at concentrations below 100 µg/mL, but above this value it begins to show cytotoxic activity. Our results may indicate that risedronate sodium, despite its strong apoptosis-inducing effect in both tested cell lines, is unable to overcome the cytoprotective effect of metamizole at 50 µg/mL, which undermines the point of the joint administration of these drugs.

The combination of 100 µg/mL of risedronate sodium and 50 µg/mL of metamizole reduced the number of cells in the G0/G1 phase, causing cell cycle arrest in the G2/M phase. This study proved that risedronate sodium slightly increases the number of the cells in the G0/G1 phase due to cell arrest, and causes a significant decrease in cell percentage in the S phase. The addition of the metamizole (50 and 5 µg/mL) caused the number of cells in the G0/G1 phase to decrease, and increased the cell number in other phases, in the cases of both the canine and human osteosarcoma cell lines. Simultaneously, the combination of 0.5 µg/mL of metamizole with 100 µg/mL of risedronate sodium did not differ statistically significantly from the results achieved in the control group. A similar effect of metamizole was observed in our studies on selected cytostatic drugs, i.e., doxorubicin, cisplatin, carboplatin, and etoposide [30]. Due to a dearth of literature on the effect of metamizole and risedronate sodium combinations, it seems difficult to conduct a deeper analysis without additional tests. Nevertheless, the results obtained here may constitute a basis for further studies. OSA therapy, both in humans and animals, is associated with numerous medical challenges that must be solved in the future. The quality of patient life seems to be of greatest importance. Even in palliative care, it is crucial to make use only of beneficial interactions between administered drugs, without any side effects diminishing therapy efficiency or simply enhancing the pathological process dynamics.

## 5. Conclusions

The influence of metamizole on cell viability in the tested lines, as well as its cytoprotective and anti-apoptotic effects, undermine its routine use in the treatment of canine and human osteosarcoma. Both alone and in combination with other drugs, including risedronate sodium, metamizole administration can bring about ambiguous effects. It seems more reasonable to choose another anti-inflammatory and analgesic drug from the analgesic ladder, as further studies are needed to explain the complex activity of metamizole.

## Figures and Tables

**Table 1:** Drug concentrations used to estimate EC_50_ value.

	Drug Name	
	Risedronate Sodium (rd)	Metamizole (meta)
Concentration (µg/mL)	300	50
	150	20
	100	10
	30	5
	15	1
	10	0.5
	3	0.1
	1.5	
	1	

**Table 2:** EC_50_ values for the tested drugs.

EC_50_ (µg/mL)		
Drug Name	D-17	U-2 OS
Risedronate sodium	144.83 ± 6.22 µg/mL	98.1 ± 5.4 µg/mL
Metamizole	>100 µg/mL	>100 µg/mL

**Table 3:** Combinations of metamizole and risedronate sodium used in the study.

Drug Combinations (µg/mL)
100 rd + 50 meta
100 rd + 5 meta
100 rd + 0.5 meta

## Data Availability

The original contributions presented in the study are included in the article, further inquiries can be directed to the corresponding author.
